# Round the Hospitals

**Published:** 1919-06-14

**Authors:** 


### ROUND THE HOSPITALS.

THE KING has been pleased to award the Royal Red Cross to the following ladies of the Nursing Services in recognition of their valuable services with the British Forces:—*Mesopotamia*: Bar to the Royal Red Cross, Assistant Matron E. R. Collins, R.R.C.; Matron F. M. Hodgins, R.R.C.; Matron E. MacFarlane, R.R.C.; Sister J. P. Stow, R.R.C.; Assistant Matron M. Walker, R.R.C. Royal Red Cross, First Class, Assistant Matron M. M. Bate, A.R.R.C.; Sister A. C. Brumwell; Matron G. Burke Roche; Sister E. Cooke; Sister O. A. Creed, A.R.R.C.; Sister I. Hay Drummond; Assistant Matron S. L. Hatton, A.R.R.C.; Sister E. Isaac, A.R.R.C.; Sister A. Leech, A.R.R.C.; Sister E. O. Marshall, A.R.R.C.; Sister N. L. Sibley, A.R.R.C.; Sister E. Smithies; Sister M. F. Valentine; Sister D. West, A.R.R.C. Second Class, Staff Nurse E. G. Adams; Staff Nurse G. Boshell; Sister M. L. Bradshaw; Staff Nurse E. I. Brookes; Sister M. Butler; Staff Nurse E. M. Carlton; Sister E. W. Dearle; Staff Nurse E. Disner; Staff Nurse W. M. Edward-Jones; Staff Nurse M. G. Etchell; Staff Nurse J. Hermon; Staff Nurse G. B. Kruger; Sister M. V. Levey; Staff Nurse K. E. Leyden; Staff Nurse H. MacHardy; Sister A. M. MacIntock; Sister L. May; Sister D. P. Meaney; Staff Nurse N. C. Packenham-Walsh; Sister C. Sinclair; Sister M. C. Trowell; Sister A. S. Walker; Staff Nurse E. J. Wells; Sister D. Wood. *Italy*: Bar to the Royal Red Cross, Principal Matron M. Steenson, R.R.C. Royal Red Cross, First Class, Sister M. Cockshott, A.R.R.C.; Sister K. M. Latham, A.R.R.C.; Assistant Matron M. E. Stewart, A.R.R.C. Second Class, Staff Nurse J. Crawford; Assistant Nurse M. W. I. Heaton-Ellis; Assistant Matron L. E. James, M.M.; Sister B. Powell-Jenkins; Sister E. V. Hutt. *East Africa*: Royal Red Cross, First Class, Assistant Matron E. L. Kelmsley, A.R.R.C.; Sister M. MacDevitt, A.R.R.C.; Matron F. Spindler. Second Class, Sister G. A. Fuller, Sister E. Spencer; Staff Nurse K. Thompson; Sister C. Tracey-Smith; Staff Nurse M. M. Wood; Staff Nurse D. L. Bishop; Sister A. E. Lowe; Sister M. Roberts; Sister A. Rose-Innes; Sister F. Walbrugh.

AMONG the honours awarded by the King on the occasion of His Majesty's birthday are the Royal Red Cross, First Class, to nursing sisters of the Royal Air Force: Matron C. Cameron, Matron L. M. Holroyd. Royal Red Cross, Second Class —*Union of South Africa*: Nurse L. M. Blackburn, Sister D. Hamilton, Sister A. Louch, Nurse A. K. Philpott. *Gibraltar*: Sister A. E. Betts. *Newfoundland*: Nurse-in-Charge J. Edgar, Nurse-in-Charge E. Reid. The King made the following awards for services in *Salonika*:—Royal Red Cross, First Class: Assistant Matron W. A. Attenborough, A.R.R.C.; Assistant Matron E. C. Ellis, A.R.R.C.; Assistant Matron A. L. Fielding; Assistant Matron M. E. Mee; Assistant Matron S. H. Mitchell; Matron M. H. Weale; Sister C. Sorenson, A.R.R.C.; Head Sister E. C. L. Wilson. Royal Red Cross, Second Class: Sister N. M. Buckley, Staff Nurse A. Cameron, Sister A. Clarke, Staff Nurse A. Cole, Staff Nurse M. E. Dixon, Nurse C. J. R. Douglas, Sister M. Drury, Nurse L. A. Duncan, Sister A. E. A. Foreman, Staff Nurse G. Fozard, Sister M. H. Gammie, Sister H. Gillespie, Sister N. B. Hutchison, Sister M. M. L. Johns, Staff Nurse A. Maguire, Staff Nurse E. McVeigh, Sister A. E. Moffat, Staff Nurse E. Moore, Sister A. I. Murray, Sister H. Powell-Evans, Sister D. M. Sempers, Staff Nurse E. S. Shewan, Sister H. E. Stacey, Sister A. Stevenson, Assistant Matron A. P. Thomson, Sister M. Upton, Sister E. A. Williams, Sister E. Winkle, Sister E. M. Bolton, Sister J. Brydon. *Egypt*:—Bar to the Royal Red Cross: Matron E. A. Cox, R.R.C.; Assistant Matron K. F. G. Skinner, R.R.C. Royal Red Cross, First Class: Sister J. M. McCarthy, A.R.R.C.; Assistant Matron N. Stewart; Sister E. E. Wraxall, A.R.R.C.; Matron J. R. Gemmell. There are also sixty-two recipients of the Second Class of the Royal Red Cross for services in Egypt.

AT the Investiture held on June 7 the King personally conferred decorations on members of the Nursing Services as follows: Bar to the Royal Red Cross, Matron Mabel Tunley, Q.A.I.M.N.S. Royal Red Cross, Second Class, Sister Alice Fitzgerald, Q.A.I.M.N.S.R., Assistant Matron Jeannie Lindsay, Sister Emma Clark, Sister Annie Elliott, Sister Hannah Henstock, and Sister Jeannie Holford, T.F.N.S.; Matron Margaret Money, Matron Bessie Stevens, Matron Alice Wellicome, and Sister Sarah Page, B.R.C.S.; Sister Sarah Demestre, Sister Elsie Greig, Sister Elsie Pidgeon, and Sister Ethel Smith, Australian A.N.S.; Nurse Freda Villiers, V.A.D. These ladies were afterwards received at Marlborough House by Queen Alexandra.

THE QUEEN, accompanied by Princess Mary, paid an informal visit on June 7 to Lord Leverhulme's Hospital at Cedar Lawn, Hampstead, which gave immense pleasure to patients, nurses, and helpers. Tea was in progress in the garden, and Her Majesty made the occasion a very happy one for all by the gracious spirit of enjoyment she brought with her. The invalids able to stand formed up with the nurses to offer a cordial farewell to the Royal visitors.

THE Edith Cavell Homes for Nurses have saved the situation as regards many home-coming sisters, and the illustrious lady whose dream it was to found them would be well content could she know into what wise hands her scheme has fallen. The chairman of the Council estimates that for the next year or two accommodation ought to be found for 1,200 nurses who are annually in need of rest. A great extension of the system of lending furnished

**ROUND THE HOSPITALS**—*(continued).*

houses to the Council would best meet this need. At present there are six homes in different localities capable of accommodating some 600 nurses a year, or about half the number who require this aid. None but nurses themselves know how unsuitable the average boarding-house is to their needs. Most of the sisters gladly pay the cost of their maintenance, and all thoroughly enjoy the delightful atmosphere and surroundings of the Homes. Father Bernard Vaughan, preaching on behalf of them at Farm Street Church lately, evoked a liberal response, the offertory amounting to £88.

The Council of the Edith Cavell Homes would confer a public benefit on the nursing service if they would undertake the provision of a sanatorium for nurses afflicted with tuberculosis. Military nurses are now put on the same footing as officers in respect of tuberculosis contracted or aggravated by their war duties. If the disease has been contracted on duty they are entitled to an allowance of £4 14s. 6d. for sanatorium treatment. In other cases a payment of £3 3s. a week is made. A sanatorium for nurses in the earlier stages of the disease ought to offer some form of training for open-air occupations. A nurse who has once had symptoms of this disease is unquestionably faced with the necessity for changing her profession, and nothing is more likely to stimulate her recovery than an opportunity for making a fresh start.

It is fitting the first annual meeting of the College of Nursing to be held out of London should take place in Manchester. The nurses of Manchester have been accustomed to frequent meetings for years, ever since the removal of the Royal Infirmary from its old site in Piccadilly to its present one in Oxford Road. Realising the need for such gatherings, Miss Sparshott, the Lady Superintendent, gained the help of the then General Superintendent, Mr. Carnt, who obtained the willing consent of the Board of Management to the use of the Nurses' Home for meetings. Since that date (1909) meetings have been held either at the Royal Infirmary, or some other place, at first three or four times a year, and during the last six or seven years, every month. These meetings have been for educational and social purposes, and all kinds of subjects have been discussed. Speakers from the various missionary societies, from the Royal British Nurses' Association, the Nurses' Social League, the National Union of Trained Nurses, and the College have all had their innings. A strong centre of the College of Nursing is now firmly established, and a most successful annual meeting and conference are anticipated. Full particulars of these fixtures, which are to take place on Wednesday and Thursday, June 18 and 19, will be found on p. 278.

An interesting letter from Miss Monk, matron of the London Hospital, on the subject of "Sick Children's Nurses," appears in the *Times* of June 6 in reply to one from Mr. John Murray. The latter gave the impression that he advocated the employment of nurses whose only training had been gained in children's hospitals, and that he considered them far more efficient for the care of sick children than nurses trained in general hospitals with special wards for sick children. Miss Monk contends that "the adult trained nurse" is in a better position all round for her duties, that she has a wider outlook and a wider character, besides many special technical advantages. Most trained nurses will agree with this point of view, and would deprecate the recognition of women who had served solely in children's hospitals as fully-trained nurses even for their own limited field of work. But in our judgment "adult nurses" are liable to undervalue the remarkable intuitive skill in dealing with infants and young children which is developed by years of patient observation and experience among them. It is impossible in the course of three or four months' work in a children's ward to learn the inner meaning of those slight changes of expression whereby an infant can throw out an appeal. The nursing of sick children is a highly specialised calling, and those who pursue it should not grudge to spend extra years in mastering its mysteries as well as in perfecting their general knowledge of diseased conditions.

In a letter to the *Times* on June 10, Mr. John Murray corrects Miss Monk's impression of his views. He states that at the Hospital for Sick Children, Great Ormond Street, the nurses who hold responsible positions are adult trained, and explains that he has no intention of depreciating the value of general training for sick children's nurses. The point at issue between Miss Monk and Mr. Murray seems to resolve itself, therefore, into one of precedence. And although theoretically it may be more consistent to begin with general training, administratively it is found much more convenient for a nurse to take training in a children's hospital first, and at an earlier age than the minimum prescribed by general hospitals.

Now that rationing has almost come to an end the country may well bear testimony to the organising ability displayed over it. It certainly abolished the queues, which are said to have engulfed a million and a-quarter people a week for long unprofitable hours. It cost, it seems, but 10d. a head a year to run, including the provision of the two books of coupons. It kept discontent from the door by equalising the lot of rich and poor. And it relieved the heads of institutions from a carking load of care in respect of the large populations for whom they had to cater. Some amount of food control has come to stay. And this might well be extended in certain directions, notably in the interests of a pure milk-supply. The dirtiness of hospital milk has been recently proved by tests applied to samples of milk from twenty-one London hospitals. Every one contained the bacillus coli, and in eight of them the bacteria per cubic centi-

**ROUND THE HOSPITALS—(*continued*).**

metre averaged 34,000,000. In the United States they have succeeded in reducing the bacteria in Grade A milk to 10,000 per cubic centimetre, which seems quite right.

THE Devonshire County Nursing Association, which now numbers 117 affiliated societies, is badly hampered for lack of money and nurses. The Reverend Prebendary Buckingham, who took the chair at the annual meeting, complained that the response to their appeals for women to take training as village nurses and midwives had been most disappointing. It would seem, from the remarks made later on, that the Association has not yet squarely tackled the question of a nurse's minimum salary, as some county associations have done to their credit and advantage. An understanding as to salaries is foreshadowed in co-operation with the Child-Welfare Committee of the County Council, and when this weighty matter is settled the Devonshire women will know better what to expect in undertaking to get trained. The contributions of the Association to the Nurses' Pension Fund involved an expenditure of £224.

A VERY important decision was arrived at when the West Riding Nursing Association held its annual meeting at Leeds under the chairmanship of the Lady Mayoress, Mrs. A. E. Hartley. It was reported that twenty-six new districts had been affiliated, and that there were over 500 rural districts in the area. The work, in fact, has grown beyond the compass of a voluntary agency, and from now on will be grafted on to that of the West Riding County Council. We should like to have fuller particulars of the scheme through which this affiliation is to be brought about. There can be no doubt that the financial strain of furnishing nursing and maternity aid to widely scattered areas is beyond the scope of a voluntary organisation unaided by grants from a central body. But it would be a calamity if voluntary effort were weakened or wiped out by this amalgamation, and we trust the well-known business ability of Yorkshiremen and Yorkshire women to see that this does not happen.

WE learn with pleasure that, owing to the constant increase of work in the Queen Victoria Nursing Institution at Wolverhampton, an extension of the District Nurses' Home is in contemplation. The Chairman, Mr. W. R. Wilson, appeals for £2,000 for this purpose, and will not appeal in vain to generous Wolverhampton.

SIR JOHN LYNN THOMAS, C.B., C.M.G., is a great believer in the future of the V.A.D. He wants to have a Welsh National Register of all V.A.D. helpers compiled through the county directors, and he believes that they could be utilised to assist village nurses in the country and Jubilee nurses in towns. He believes also that they might be drawn upon to meet the lamentable deficiency of private nurses. He suggests that the V.A.D. could make observations on the rate and condition of the pulse, the rate and character of respiration in chest and abdominal diseases, the site and intensity of pain, and the condition of the extremities, and could report these facts to the trained nurse, who would report them to the doctor. We regret to say that we are entirely sceptical about the value of this chain of observation. We do not know of any voluntary war nurses who want to go on acting after the manner indicated, and, if we did know any, we should certainly advise them to take training and learn their business satisfactorily.

THE Royal Devon and Exeter Hospital was *en fête* on May 29, when the annual awards of prizes and certificates were made at the hands of Lady Chaning Wills, wife of the President. The gold medal was won by Nurse Barnes, with 948 marks, Nurse Beddowe, who won the silver medal, having 903. Nine nurses obtained certificates. Mr. Domville, the examiner, spoke warmly of the help given by Sister Baron in training the probationers. Lady Wills, in alluding to the excellent administration which had triumphed over adverse circumstances, spoke of the need for extending the nurses' quarters, for which an appeal has been circulated. A bouquet of sweet peas was presented to the matron, and one of lilies of the valley to Lady Wills.

THE Local Government Board of Scotland has long been far ahead of England in respect of the education of nurses. Early in May the usual examination was held for the certification of trained sick nurses and trained fever nurses in Glasgow, Edinburgh, Dundee, and Aberdeen. The four male examiners were assisted in the practical part by Miss Merchant, Matron of the Eastern District Hospital, Glasgow, and Miss Lindsay, Matron Belvidere Hospital, Glasgow. The subjects for examination were elementary anatomy and physiology, hygiene and dietetics, medical and surgical nursing, midwifery (for Poor-Law and general-trained nurses only), and infectious diseases (for fever-trained nurses only). The number of candidates who presented themselves was 408. Of these twenty-nine have now received the certificate of general training and efficiency granted by the Board, subject to the satisfactory completion of three-years' training in hospital, and 111 have gained the certificate in fever training. The proportion of failures seems very large, and seems to point to the need for trained sister tutors in these establishments.

BEST congratulations to the Elizabeth Garrett Anderson Hospital on the progress of their Endowment Fund during these late lean years. Courageously they set about raising £50,000 in 1916, and of this amount nearly £44,000 has been already subscribed, principally by women and girls. There remains some £6,000 to be got in this summer.

